# Which factors influence the quality of end-of-life care in interstitial lung disease? A systematic review with narrative synthesis

**DOI:** 10.1177/02692163211059340

**Published:** 2021-12-17

**Authors:** Evelyn Palmer, Emily Kavanagh, Shelina Visram, Anne-Marie Bourke, Ian Forrest, Catherine Exley

**Affiliations:** 1 Royal Victoria Infirmary, Newcastle upon Tyne, UK; 2 Marie Curie Hospice Newcastle, Newcastle upon Tyne, UK; 3 Newcastle University, Population Health Sciences, Newcastle upon Tyne, UK

**Keywords:** Interstitial lung disease, palliative care, end-of-life care, death, systematic review

## Abstract

**Background:**

People dying from interstitial lung disease experience considerable symptoms and commonly die in an acute healthcare environment. However, there is limited understanding about the quality of their end-of-life care.

**Aim:**

To synthesise evidence about end-of-life care in interstitial lung disease and identify factors that influence quality of care.

**Design:**

Systematic literature review and narrative synthesis. The review protocol was prospectively registered with PROSPERO (CRD42020203197).

**Data sources:**

Five electronic healthcare databases were searched (Medline, Embase, PubMed, Scopus and Web of Science) from January 1996 to February 2021. Studies were included if they focussed on the end-of-life care or death of patients with interstitial lung disease. Quality was assessed using the Critical Appraisal Skills Programme checklist for the relevant study design.

**Results:**

A total of 4088 articles were identified by initial searches. Twenty-four met the inclusion criteria, providing evidence from 300,736 individuals across eight countries. Most patients with interstitial lung disease died in hospital, with some subjected to a high burden of investigations or life-prolonging treatments. Low levels of involvement with palliative care services and advance care planning contributed to the trend of patients dying in acute environments. This review identified a paucity of research that addressed symptom management in the last few days or weeks of life.

**Conclusions:**

There is inadequate knowledge regarding the most appropriate location for end-of-life care for people with interstitial lung disease. Early palliative care involvement can improve accordance with end-of-life care wishes. Future research should consider symptom management at the end-of-life and association with location of death.

What is already known about the topic?

- The majority of patients with interstitial lung disease die in hospital.
- Patients with interstitial lung disease experience a high symptom burden which escalates towards the end-of-life.
- The uncertain disease trajectory in interstitial lung disease contributes to late referral to palliative care services.

What this paper adds?

- This study confirms low levels of referrals to specialist palliative care services and engagement with advance care planning amongst patients with interstitial lung disease.
- When preferred place of death is ascertained, many patients wish to die at home. However, the majority of patients with interstitial lung disease die in hospital.
- There is a paucity of research that considers symptom control at the end-of-life in this patient group.

Implications for practice, theory, or policy

- It is important to recognise the uncertain trajectory seen in interstitial lung disease and refer patients to specialist palliative care services early in the disease process to enable advance end-of-life care planning interventions.
- Services should be configured to support patients dying at home if this is their preference for care.
- Further research is needed to assess symptom control at the end-of-life in both hospital and community settings to review the safety and appropriateness of dying at home with interstitial lung disease.

## Introduction

Interstitial lung diseases are a heterogenous group of conditions characterised by inflammation and fibrosis of the lung parenchyma.^1,2^ The most prevalent symptoms are severe breathlessness, cough, fatigue, anxiety and depression.^3^ Irreversible fibrosis and respiratory failure develop in the final stages of many forms of interstitial lung disease.^4^ Idiopathic pulmonary fibrosis is the most common type of interstitial lung disease to cause progressive fibrosis and is associated with a median prognosis of 3 years.^5^

Disease trajectory in advanced interstitial lung disease can be unpredictable, with a background depletion of lung function and symptomatic decline which is punctuated by acute exacerbations of symptoms.^6^ An acute exacerbation has a significant impact on patients’ prognosis, with up to 50% of patients dying during an exacerbation.^7^ National guidelines recommend early involvement of specialist palliative care services in the management of patients with interstitial lung disease.^5^ A palliative care approach can help with symptom management, facilitate discussions about the future and end-of-life care, and improve patients’ satisfaction with their care.^8^

Previous systematic reviews have considered end-of-life care for patients with lung cancer and chronic obstructive pulmonary disease (COPD).^9,10^ However, patients with advanced interstitial lung disease follow a more uncertain disease trajectory compared with patients with advanced cancer, leading to problems with initiating timely palliative care.^6,11^ This review aimed to (i) identify studies involving people with interstitial lung disease who were approaching the end-of-life or had died and (ii) synthesise evidence about the quality of their end-of-life care.

## Methods

### Study design

This was a systematic review of existing literature. The study protocol was prospectively registered with PROSPERO (PROSPERO 2020 CRD42020203197 https://www.crd.york.ac.uk/prospero/display_record.php?ID=CRD42020203197). Narrative synthesis was implemented due to the heterogenicity of included studies, using guidance published by the Economic and Social Research Council (ESRC).^12^ Reporting was performed according to the PRISMA (Preferred Reporting Items for Systematic Reviews and Meta-Analyses) guidelines.^13^

### Search strategy

**Figure 1.**
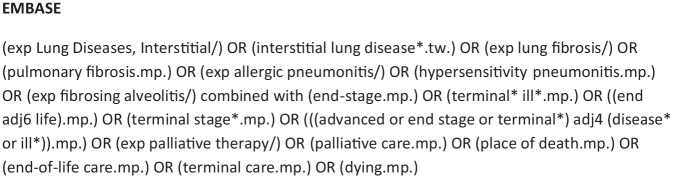
Example search strategy for the systematic review.

Five electronic databases were searched (Medline, Embase, PubMed, Scopus and Web of Science) from 1st January 1996 to 28th February 2021. The search strategy was constructed with input from a medical librarian (Figure 1). The date range was limited to articles published after 1996 as the management of interstitial lung disease and end-of-life care has changed substantially in the last 25 years and the aim was to focus on contemporary literature. No exclusions were made based on country of publication, although only studies published in English were included.

### Study inclusion criteria

Studies that reported quantitative data related to the quality of end-of-life care or death of individuals with interstitial lung disease were included. The UK General Medical Council’s definition of end-of-life care was adopted; this describes end-of-life care being in the last 12 months of life.^14^ Qualitative studies were excluded as this was felt to be beyond the scope of this analysis and would have contributed to already substantially heterogeneous data. A separate review was conducted which concentrated on qualitative evidence relating to palliative care in interstitial lung disease. No other restrictions were placed on the study designs eligible for inclusion. Review articles, conference proceedings, editorials and opinion letters were excluded. Studies focussed on a specific intervention (e.g. opioids) at the end-of-life were excluded as they did not provide information about the overall quality of end-of-life care.

The included studies considered all types of interstitial lung disease. Studies that focussed on patients awaiting a lung transplant were excluded, as these patients often follow a very different end-of-life trajectory. Studies that included data on individuals with other chronic lung diseases were included if results were presented separately for individuals with interstitial lung disease.

### Data collection

Database search results were exported and merged in an EndNote library. After removing duplicate studies, two researchers (EP and EK) independently screened the titles and abstracts of the retrieved studies for eligibility. Full texts were obtained and reviewed for studies that were thought to meet the review criteria. These were reviewed by both researchers to determine study inclusion and any disagreements were resolved by consensus. The reference lists of all included studies were searched for additional studies that met the review criteria.

### Quality assessment and risk of bias

Two researchers (EP and EK) independently assessed the methodological quality of included studies using the relevant Critical Appraisal Skills Programme (CASP) checklists. Based on these tools an assessment of the methodological quality was made as ‘Good’, ‘Fair’ or ‘Poor’ (see Supplemental material). No studies were excluded based on the methodological assessment; however, the quality assessment was used as a variable in the subsequent analysis and studies with ‘poor’ methodology were given less weighting in the analysis.

### Data analysis and synthesis

Meta-analysis was not possible due to the heterogeneity in the design and reported outcomes of the included studies. Data analysis was performed (by EP) using the four-stage approach described by Popay et al.^12^ In the initial stage, a theoretical model was developed based on the results of the scoping literature search which informed decisions about what type of studies to include. The second stage involved the development of a preliminary synthesis by grouping the included studies based on their reported outcomes to allow description of patterns and enable factors which influenced end-of-life care to be identified across the studies. The third stage explored relationships within and across the included studies to understand how and why factors influenced end-of-life care. Vote counting based on direction of effect was used to synthesise results as the type of effect measure varied across the studies. To support interpretation, provide clarity and aid visual representation the data were organised into a data matrix. The final stage assessed the robustness of the synthesis by undertaking an overall assessment of the strength of evidence available for drawing conclusions. Studies with a high risk of bias were given less weight in the synthesis.

## Results

### Study characteristics

**Figure 2.**
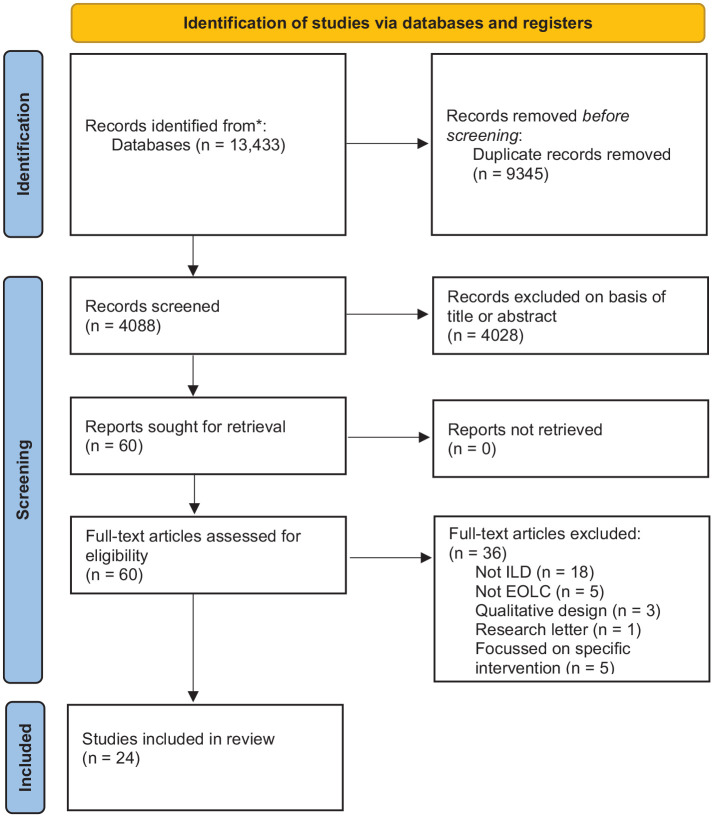
Adapted from Preferred Reporting Items for Systematic Review and Meta-Analyses (PRISMA) 2020 flow diagram. ILD: interstitial lung disease; EOLC: end-of-life care.

The search identified 4088 publications, resulting in 60 full text articles reviewed. Of these, 24 studies were included in the final analysis (Figure 2; Table 1). The majority of included studies (14/24) were retrospective observational designs; three presented survey data; and three were retrospective case-controlled cohort studies. The review included one prospective cohort study and three studies identified as randomised controlled trials. Two studies also captured qualitative data from semi-structured interviews; these data were not included in this analysis.

**Table 1.** Characteristics of included studies and quality assessment score.

Author	Aim of study	Population (country)	Study design	Quality assessment	Key findings of the study
Ahmadi et al.^23^	Compare the prevalence of symptoms and palliative treatments between patients dying of oxygen dependent ILD and lung cancer	ILD *n* = 285 lung cancer *n* = 10,822 (Sweden)	Retrospective analysis of clinical database	Fair	ILD patients have high symptom burden and more likely to have unrelieved symptoms of breathlessness, pain, and anxiety Death is more likely to be ‘unexpected’ for ILD patients (15% vs 4%; *p* < 0.001) and less likely to occur in a palliative setting (17% vs 48%) EOL discussions with patients were less common than in lung cancer (41% vs 59%; *p* < 0.001) ILD patients access fewer palliative care services compared with patients with lung cancer
Akiyama et al.^30^	Characterise the practice of pulmonologists in relation to palliative care and EOL communication. Identify barriers to providing palliative care	Respiratory physicians *n* = 130 (views regarding IPF patients) (Japan)	Self-administered survey	Fair	Physician associated barriers significantly associated with difficulty providing palliative care: few established treatments and difficulty predicting prognosis (*p* = 0.04), discrepancies in understanding and care goals between patients, families and physicians (*p* = 0.03), inadequate communication about the goal of care (*p* = 0.003) Physicians found it more difficult to provide palliative care for patients with IPF than those with lung cancer (*p* < 0.001) Physicians were less likely to prescribe opiates for patient with IPF than those with lung cancer (*p* < 0.001) EOLC discussions were held later than the physician perceived optimal timing (*p* < 0.001)
Archibald et al.^27^	Explore the effects of palliative care and other factors on location of death	ILD *n* = 92 (Canada)	Retrospective review of patient electronic records	Good	Home and hospice deaths are feasible in ILD with appropriate MDT support Early initiation of palliative care components such as ACP discussions, symptom self-management, caregiver engagement and close collaboration with allied health care professionals can improve adherence to preference for home or hospice death 62% patients died at home or hospice
Bajwah et al.^17^	Assess the palliative care needs of fibrotic ILD patients	ILD *n* = 45 (UK)	Retrospective review of patient electronic records	Fair	93% patients experienced breathlessness in last year of life. Other symptoms included cough, fatigue, depression/anxiety, and chest pain. 76% died in an acute setting All patients who received opioids (49%) or benzodiazepines (18%) for symptoms had a documented benefit (100% documented benefit) Non-pharmacological interventions to manage symptoms are seldom used
Bajwah et al.^18^	Assess the impact of a case conference delivered at home and evaluate the feasibility and acceptability	IPF *n* = 53 (UK)	Randomised controlled trial Qualitative interviews	Poor	Case conference intervention associated with positive effect on palliative care concerns, QoL, anxiety and depression Improvement in Palliative Care Outcome Scale (POS) score −5.7 following case conference
Barrat et al.^33^	Assess the effectiveness of a novel MDT approach to assess the palliative care needs of patients with ILD	ILD *n* = 72 pre-MDT cohort, *n* = 26 post-MDT cohort, *n* = 46 (UK)	Case controlled study	Good	Increased documentation of DNAR discussions (pre-MDT 38.5%, post-MDT 78.3%, *p* < 0.001) Increased referral to specialist palliative care services (pre-MDT 38.5%, post-MDT 73.9%, *p* < 0.01) Increased referrals to hospice delivered fatigue and breathlessness course (pre-MDT 30.8% post-MDT 67.4%, *p* < 0.01)
Barril et al.^31^	Determine the current situation in palliative care for patients with ILD in Spain	Healthcare professionals (majority respiratory physicians) *n* = 164 (views regarding ILD patients) (Spain)	Self-administered survey	Poor	46% respondents had formal training in palliative care, 56% relied on SPC teams to deliver palliative care 63% respondents discussed EOL process with their patients, 78% discussed limitations of life sustaining treatments, 22% discussed preferred place of death Identified a lack of palliative care protocols and training
Brown et al.^19^	Explore the differences in the palliative care of patients with ILD and COPD who die in ICU compared with patients with cancer.	ILD *n* = 79 COPD *n* = 592 Metastatic cancer *n* = 158 (USA)	Retrospective review of patient electronic records	Fair	Patients with ILD and COPD receive fewer elements of palliative care and have a longer length of stay than patients with cancer. ILD patients were less likely to have DNAR in place at time of death compared with patients with cancer (OR 0.40; 95% CI 0.19–0.86) ILD pts less likely to have assessment of pain in last 24 h of life (OR 0.43; 95% CI, 0.19–0.97)
Cross et al.^38^	Determine the trends and factors associated with place of death among individuals with chronic lung disease	ILD *n* = 246,210 COPD *n* = 2,026,758 CF *n* = 6907 (USA)	Retrospective review of national death registry database	Good	Home deaths are increasing among decedents from chronic lung disease Dying at home was more likely for patients living in rural areas, were male, married, higher level of education Patients with ILD and CF were more likely to die in hospital (ILD 48%) than patients with COPD Low numbers of patients with ILD died at home (28%) and in hospice (5%) Rate of hospital deaths in ILD reduced by 23% between 2003 and 2017
Guo et al.^16^	Examine the management approach and diagnostic test burden for ILD patients in their final admission	ILD *n* = 67 (Australia)	Retrospective review of patient’s medical records	Fair	57% patients died on medical ward, 22% palliative care ward, 5% ICU ACP conversations occurred for 16% prior to hospital admission Patients underwent fewer investigations if they had received outpatient palliative care (*p* < 0.001), used LTOT (*p* < 0.001) or had documented ACP (*p* < 0.004) 36% patients had last diagnostic test on the day goal of care was changed to palliation, 18% had further investigations after goal of care was changed
Higginson et al.^37^	Determine the trends and factors associated with dying in hospital with COPD and ILD	ILD *n* = 45,712 COPD *n* = 334,20 (UK)	Retrospective review of death registry database	Good	70% patients with ILD died in hospital (compared with 67% patients with COPD) Hospital deaths reduced between 2004 and 2014 by 3%, but this trend was not seen for patients with comorbidities Multimorbidity, living in urban area and deprivation increased the chance of death in hospital
Janssen et al.^35^	Assess effect of palliative care on anxiety and depression and QoL. Review the feasibility of measuring the effect of palliative care clinic referral.	IPF *n* = 22 (USA)	Randomised controlled trial	Poor	Receiving palliative care did not lead to an improvement in QoL, anxiety or depression compared to usual care Following involvement with SPC patients trended towards worsening symptoms (*p* = 0.066 at 3 months and *p* = 0.055 at 6 months) and had a transient worsening in depression (*p* = 0.008 at 3 months, did not persist at 6 months *p* = 0.26) Lung function tests were all negatively correlated with symptom, impact, and activity scores (*p* < 0.0001)
Kalluri et al.^20^	Explore the association between an early integrated palliative approach with acute care utilisation in last year of life and location of death.	IPF *n* = 32 *n* = 22 after service change *n* = 10 control (Canada)	Case controlled study	Fair	Patients who received early integrated palliative care were more likely to die at home (55% vs 0%). 4.59 times more likely to die at home/hospice. Rates of ACP improved from 40% to 100% for the patients involved with the clinic. Preferred place of death was recorded – 77% PPD Home, 14% PPD Hospice. Early integrated palliative approach reduced acute healthcare utilisation (12.3 times less likely to have emergency department visit and 2.32 times less likely to require hospital admission)
Kalluri et al.^21^	Evaluate the difference in resource use and associated costs of EOLC between patients who receive integrated palliative care and standard care	IPF *n* = 2768 *n* = 78 received intervention *n* = 2166 specialist care *n* = 524 non-specialist care (Canada)	Case controlled study	Fair	Integrated palliative care group had lower healthcare costs driven by a reduction in emergency department visits and hospitalisations Lower rates of hospital deaths in the integrated palliative care group (45%) than specialist care (65%) or non-specialist care (67%) Integrated palliative care approach has higher outpatient cost (both secondary care and GP appointments), however, as more patients supported in dying at home this reduced healthcare costs at end of life
Kim et al.^29^	Understand factors affecting decisions regarding referrals to SPC services and address barriers/facilitators to referrals	Healthcare professionals *n* = 36 (views regarding ILD patients) (UK)	Self-administered survey	Poor	Barriers to SPC referral – concern about continuity of care and patients feeling abandoned, long waiting lists and limited beds. 57.5% felt SPC involvement was unlikely to add anything to patients’ care Triggers for referral; EOLC, general deterioration, poorly controlled symptoms, hospital admission.
Koyauchi et al.^28^	Are there differences in the quality of dying and death (QODD) and end of life interventions between patients with ILD and those with lung cancer?	ILD *n* = 177 (Japan)	Retrospective review of medical notes and survey of bereaved relatives	Good	Patients with ILD were more likely to experience breathlessness at the end of life than those with lung cancer (94.8% vs 84.9%; *p* = 0.043) Patients with ILD had significantly lower Good Death Index scores for QODD than those with lung cancer (4.33 vs 4.57; *p* = 0.04) Opioid administration in the last hospital admission was lower in patients with ILD than those with lung cancer (0.6% vs 32.1%; *p* < 0.001). Patients with ILD were less likely to be referred to SPC (8.5% vs 54.3%; *p* < 0.001) Patients with ILD underwent more investigations in their terminal admission than patients with lung cancer
Liang et al.^18^	Describe the frequency and characteristics of patients with IPF who were admitted to ITU and frequency of referrals to palliative care	IPF *n* = 106 (USA)	Retrospective cohort	Fair	77% patients died during ITU admission. 84% patients were admitted for acute respiratory deterioration 25% patients were referred to SPC, only 4% had palliative care referral prior to ITU admission Median time from first specialist clinic visit to ITU admission was 9.5 months (range 0–83 months), 36% admitted before second clinic visit and 47% prior to third clinic visit
Lindell et al.^36^	Assess the impact of a nurse delivered support group on the HRQoL of patients with IPF and their carers	IPF *n* = 42 (patients and carers) (USA)	Randomised controlled trial Qualitative interview	Poor	Anxiety reported by 58% patients Lower HRQoL scores (*p* = 0.038) and trend towards increased anxiety (*p* = 0.077) for patients following intervention No impact on anxiety/depression or HRQoL for carers following intervention, but positive impact on perceived stress (*p* = 0.018) Qualitative themes suggested feelings of less isolation, a more balanced view on illness and personal satisfaction from being involved in research
Lindell et al.^22^	Describe the time course of events prior to death in patients with IPF at tertiary centre.	IPF *n* = 404 known location of death *n* = 277; unknown location of death *n* = 127 (USA)	Retrospective review of clinical database	Fair	57% patients died in hospital Low rates of palliative care consultation (13.7%) and this was more likely if patients were in ICU (34%) or hospice (47%) Palliative care referrals occurred late in the disease process (<1 month prior to death in 71%) Average delay of 2 years from time of diagnosis to referral to specialist centre
Rajala et al.^15^	Describe treatment practices, decision making and symptoms during EOLC	IPF *n* = 59 (Finland)	Retrospective review of patient electronic records	Fair	80% patients died in hospital, 14% at home Life prolonging treatments were continued until last days of life (more likely if patients died in tertiary hospital vs community hospital *p* < 0.05) During their final hospital admission most patients received antibiotics (79%) NIV treatment was given to 36% EOL and resuscitation decisions were made late – 24% DNAR initiated <3 days prior to death Opiates prescribed for 71% patients and anxiolytics for 44%
Rajala et al.^34^	To evaluate IPF patients’ symptoms and HRQoL over last 2 years of life	IPF *n* = 247 (Finland)	Prospective cohort study Self-administered questionnaires	Good	Rapidly increased impairment of HRQoL and escalating symptom burden in last 6 months of life Exceptionally low HRQoL and severe breathlessness and fatigue reported 2 years prior to death
Rush et al.^17^	Attempt to characterise the utilisation of palliative care referrals in patients with IPF undergoing mechanical ventilation	IPF *n* = 3166 (USA)	Retrospective analysis of national database	Fair	12.9% received palliative care input Palliative care utilisation increased almost 10-fold between 2006 and 2012 (2.3%–21.6%) Increased access to palliative care associated with increasing age (*p* < 0.01), treatment at urban teaching hospital (*p* < 0.01) and DNAR status (*p* < 0.01)
Smallwood et al.^26^	To examine the care delivered to patients with fibrotic ILD during the terminal hospital admission and the past 2 years of life	ILD *n* = 67 (Australia)	Retrospective review of patients’ medical records	Good	Only 6% patients accessed specialist ILD service and 1% accessed integrated respiratory and palliative care clinic 36% received SPC input in the 2 years prior to death During their terminal admission 10 (15%) patients were admitted under SPC. A further 33 (49%) patients were referred to SPC, on average 1 day prior to death
Zou et al.^25^	Describe the patient and clinical factors associated with SPC referral and the impact of this on mortality and location of death	IPF *n* = 828 (USA)	Retrospective review of clinical database	Fair	57% patients died in hospital Palliative care referrals made for 44% patients. Palliative care referral was associated with higher chance of dying at home (OR 0.52, 95% CI 0.31–0.86, *p* = 0.010) Palliative care referral was more likely in older patients (*p* < 0.001), increased co-morbidities (*p* = 0.011), and this cohort lived closer to the hospital, were more active in support groups and had increased number of outpatient appointments (*p* < 0.001) Palliative care referral rates increased between 2000–2012 and 2012–2016 (11.5%–21.4%) which was associated with the introduction of a palliative care clinic

EOL (C): end of life (care); SPC: specialist palliative care; HRQoL: health-related quality of life; MDT: multidisciplinary team; DNAR: resuscitation order; ACP: advance care planning; PPD: preferred place of death.

Most studies included patients with interstitial lung disease (21/24), with 12 focussing on patients with idiopathic pulmonary fibrosis and five including other patient groups (COPD, cancer, cystic fibrosis). Three studies presented survey data from healthcare professionals, one included patients with idiopathic pulmonary fibrosis and their carers and one included survey data from bereaved carers.

### Quality assessment

The majority (17/24) of the studies involved retrospective designs and therefore have potential for record bias. Four studies reviewed large national databases, which increases the risk of coding errors. Sample size ranged from 22 to 246,210 participants. Three studies focussed on patients in intensive care and results are therefore not likely to be generalisable to the whole population.

Based on the CASP assessments seven studies were assessed as good quality, 12 as fair and five were poor quality (Table 1).

### Themes identified from the data

Five main factors which influenced the end-of-life care received by patients with interstitial lung disease were identified and are discussed below: health care utilisation in the last year of life, involvement with palliative care services, advance care planning, symptom management and location of death (Table 2).

**Table 2.** Summary of results: organised by themes identified from the studies.

Healthcare utilisation in the last year of life^15–21^	Hospital admission^15,16^	• 93% patients were admitted to hospital in the last year of life^15^
		• Diagnostic investigations continued until last few days of life^15,16^
		• Resuscitation decisions made late (24% <3 days prior to death)^15^
		• Decisions that the goal of care should change to ‘palliation’ were made later (median 1 day prior to death)^16^
	Intensive care^17–19^	• Leading course for emergency admission was acute respiratory failure in >80%^17,18^
		• Mortality associated with mechanical ventilation was high^18^
		• ILD patients had longer ITU stay and more likely to receive resuscitation than those with metastatic cancer^19^
	Palliative care^20,21^	• Early palliative care input was associated with reduced use of acute healthcare services and reduced costs^20,21^
Involvement of palliative care services	Referral rates^15–18,22–26^	• Wide raging rates of involvement of specialist palliative care 0%–38%^15–18,22–26^
		• Increasing referrals rates over time^17,25^
	Timing of referral^16,22,27^	• Palliative care referrals were made late in the disease process^16,22^
		• Early integrated palliative care was associated with increased adherence with PPD^27^
	Healthcare professionals’ views^29–31^	• Barriers to palliative care referral: concern that patients might feel ‘abandoned’, disruption of continuity of care, perceived long waiting lists and limited beds^29^
		• Insufficient training in palliative care^31^
		• Physicians experienced more difficulty providing palliative care to patients with IPF than those with lung cancer^30^
Advance care planning	Advance care planning uptake^15,16,18,28^	• Low rates of advance care planning discussions^15,16,18,28^
	Advance care planning interventions^20,27,32,33^	• Interventions aimed at integrating early specialist palliative care increased rates of advance care planning^20,27,32,33^
Symptom control	Symptoms of end-stage ILD^15,23,24,32,34–36^	• Most frequently reported symptom was breathlessness, reported by 66–93% patients^15,23,24,34^
		• Symptoms progressed rapidly in the final 2 years of life with a significant decline in health-related quality of life^34^
		• Mixed evidence about whether specialist palliative care involvement improved patients’ symptoms^32,35,36^
	Comparison with patients with lung cancer^23,28,30^	• Patients with ILD were more likely to experience breathlessness at end of life and had less access to palliative care than those with lung cancer^23,28^
		• ILD patients required more doses of ‘as required’ medications for symptom management with lower rates of complete relief from breathlessness^23^
		• Patients with ILD had a significantly lower mean Good Death Inventory (GDI) score than those with lung cancer^28^
		• Physicians were more likely to prescribe opioids for patients with lung cancer than IPF (<0.001)^30^
	Symptom control and the end of life^15,19,23,24,26^	• Deficiency in documented symptom assessment in ILD compared with other chronic lung diseases^19,23^
		• In the last week of life, 71%–94% patients received opioids and 44%–73% benzodiazepines^15,26^
Location of death	Location of death^15,22,24,25,37,38^	• Most patients with ILD die in a hospital setting (57–80%)^15,22,24,25,37^
		• The number of patients dying with ILD in acute settings has decreased over time^37,38^
	Factors which influence location of death^25,27,37,38^	• Death in an acute setting was associated with multimorbidity, living in urban areas and socio-economic deprivation^37^
		• Death at home was more likely for patient who were older, married, living in rural areas and who had higher level of education^25,38^
		• Earlier initiation of palliative care increased the number of patients with ILD who died in their own home or hospice^27^

### Healthcare utilisation in the last year of life

High levels of acute healthcare utilisation were reported amongst patients with interstitial lung disease towards the end of life. Studies that considered healthcare utilisation encompassed management on a hospital ward,^15,16^ in the intensive care environment^17–19^ and with input from specialist palliative care services in the community.^20,21^ Retrospective observational data from Finland reported that 93% of patients with idiopathic pulmonary fibrosis were admitted to hospital in the last 6 months of life.^15^ Another study conducted in Australia concluded that death in an acute setting was associated with increased burden of investigations and life-prolonging treatments that continued until the last few days of life.^16^ Both studies identified that many patients underwent diagnostic investigations in the final days of life (between 36% and 54% patients). Decisions about resuscitation were made late (24% <3 days prior to death)^15^ and recognition that the goal of care was ‘palliation’ occurred late (median 1 day prior to death).^16^ These data indicate that many patients are not identified as dying even in the final 24 h prior to their death and as such good end-of-life care is unlikely to be achieved. The results of both studies highlight the magnitude of acute healthcare admissions and burden of investigations which dominate the final months of interstitial lung disease patients’ lives.^15,16^

Three US studies considered the care of patients with interstitial lung disease in the intensive care environment (ITU). Unsurprisingly, the leading cause of emergency admission was acute respiratory failure in >80% patients^17,18^ and mortality associated with mechanical ventilation was high (77%).^18^ A retrospective review of patients who died within 30 days of ITU admission found patients with interstitial lung disease had longer ITU length of stay and were more likely to receive resuscitation shortly before death than patients with metastatic cancer.^19^ These data suggest that difficulty assessing prognosis led to continuation of invasive interventions and prolongation of the dying phase for patients with interstitial lung disease. However, the study did not report the diagnosis or reason for ITU admission for either the lung disease or cancer groups, which would influence both prognosis and length of stay.

A Canadian group demonstrated that the introduction of a multidisciplinary care model with early integrated palliative care and an emphasis on community-based care was associated with reduced utilisation and cost of acute healthcare services in the last year of life.^20,21^ Patients in the multidisciplinary care arm were 12.3 times less likely to require an acute emergency department visit and 2.3 times less likely to have a respiratory-related hospital admission than those who received standard management. A follow-up study assessed the impact of this multidisciplinary care model on health care costs at the end-of-life for patients with idiopathic pulmonary fibrosis.^21^ Increased outpatient and community costs were associated with the model; however, this was offset by the increase in adherence to patients’ wishes to die at home which was associated with lower health care costs.

### Involvement with palliative care services

The included studies demonstrated widely different results relating to palliative care involvement amongst people with interstitial lung disease ranging from 0% to 38%.^15–18,22–26^ Observational studies from the US reported an increase in specialist palliative care referrals over time.^17,25^ Possible reasons for the observed differences in palliative care involvement include variation in methods of data collection (review of database vs review of individual medical records), differences in clinical practice between countries and the definition used to record palliative care involvement (referral vs documentation of review in medical notes). Despite the poor prognosis associated with ventilation in interstitial lung disease, palliative care referral rates for patients in intensive care were similar to those reported in other studies, between 13% and 25%.^17,18^ Only 3% of palliative care referrals were made prior to intensive care admission and the average time from first clinic appointment to admission was 9.5 months.^18^ These data emphasise that an acute exacerbation can cause rapid and often unexpected deterioration and death in this patient population and supports the adoption of early integrated palliative care.

Studies that considered the timing of palliative care referrals reported that referrals were made late in the disease process.^16,22^ A large US observational study established that 71% of referrals to palliative care were made less than 1 month prior to death.^22^ An Australian study which focussed on inpatient management found that patients were not recognised as dying until a few days before death and that referrals to palliative care were made on average only 1 day prior to death.^16^ Previous involvement with palliative care services and advance care planning discussions were associated with a reduced burden of investigations towards the end of life (*p* < 0.001 and *p* = 0.004 respectively).^16^ Other studies demonstrated that early involvement of palliative care services (defined as >1 month prior to death) was associated with a reduction in healthcare utilisation and improved adherence with patients’ preferred place of death.^20,27^ A recent Japanese study concluded that involvement with palliative care and participation in end of life discussions improved the quality of death and dying for patients with interstitial lung disease, however, patients were less likely to be referred to specialist palliative care than those with lung cancer (8.5% vs 54.3%; *p* < 0.001).^28^

Two studies identified factors associated with increased access to palliative care services on a population level.^17,25^ Factors associated with increased palliative care referral among patients with idiopathic pulmonary fibrosis receiving mechanical ventilation were older age, resuscitation status and treatment at an urban teaching hospital.^17^ A national US study also reported increased palliative care referrals with older age and co-morbidities, as well as patients who lived closer to the hospital, engaged with support groups, and were seen in outpatient clinics more frequently.^25^ Studies that considered barriers to accessing palliative care focussed on physician-related barriers.^29,30^ A survey of UK health professionals established physician-associated barriers preventing referral to palliative care, including concern that patients might feel ‘abandoned’, disruption of continuity of care, perceived long waiting lists and limited beds.^29^ However, this was a small survey (36 respondents), limited to one area of the UK with only an approximate 15% response rate. A survey of Japanese respiratory physicians indicated that physicians experienced more difficulty providing palliative care to patients with idiopathic pulmonary fibrosis than those with lung cancer.^30^ Difficulty predicting prognosis, inadequate communication, and discrepancies in understanding of goals of treatment between patients, families and physicians were all associated with difficulty in providing palliative care. This survey included a larger number of respondents (*n* = 130) with a 96% respondent rate. A large proportion (57.7%) of respondents to the UK survey felt that specialist palliative care involvement was unlikely to add anything to their patients’ management.^29^ The majority (71.6%) of respiratory physicians responding to the Japanese survey felt there were few established treatments to relieve patients’ symptoms.^30^ These findings suggest inadequate training and awareness of palliative care. This interpretation is supported by a survey of 164 Spanish respiratory physicians that identified insufficient training in palliative care, with only 46% respondents having received specific training.^31^

### Advance care planning

In general, there were inadequate advance care planning opportunities provided for patients with interstitial lung disease,^15,16,18,28^ however, there was evidence that the integration of respiratory and palliative care services led to increased advance care planning discussions.^20,27,32,33^ An Australian study reviewing the final hospital admission for patients with interstitial lung disease reported that only 16% participated in advance care planning prior to their terminal admission, but those that had documented advance care planning decisions had fewer investigations towards the end-of-life.^16^ Death was more likely to be ‘unexpected’ in patients with interstitial lung disease compared to lung cancer (15% vs 4%; *p* < 0.001),^23^ which may affect the rates of advance care planning uptake in this patient group. A Japanese study reported that patients with interstitial lung disease were more likely to discuss ‘resuscitation’ and less likely to discuss ‘place of death’ than patients with lung cancer,^28^ suggesting that the uncertain disease trajectory in interstitial lung disease may impact some aspects of advance care planning discussions.

Four studies reported the outcome of novel approaches to collaboration between respiratory, palliative care and community teams that all resulted in increased advance care planning discussions amongst patients with interstitial lung disease.^20,27,32,33^ A community case conference devised in the UK, which supported patients and carers with advance care planning, improved symptom control and quality of life for participants.^32^ Another UK service developed a collaborative multidisciplinary team approach which led to increased advance care planning discussions and documentation of resuscitation decisions, in conjunction with referrals to community teams to address other unmet palliative care needs.^33^

### Symptom management

Patients with interstitial lung disease experienced a high burden of symptoms^15,23,24,34^ and there were deficits in their symptom management at the end-of-life when compared to patients with lung cancer.^23,28^ The most frequent symptom reported in end-stage disease was breathlessness (66%–93% of patients).^15,23,24,34^ Other commonly reported symptoms include cough, fatigue, anxiety and depression.^24^ Survey data from people with idiopathic pulmonary fibrosis in Finland established that symptoms progressed rapidly in the final 2 years of life with a significant decline in health-related quality of life.^34^

The primary aim of palliative care is to improve symptom management. Three studies considered the effect of palliative care interventions on symptom management for people with interstitial lung disease reporting varied results.^32,35,36^ A UK study reported improvement in interstitial lung disease patients’ symptom control, quality of life and anxiety and depression through involvement with a community case conference.^32^ However, these results are challenged by a recent US study which found that involvement with palliative care services transiently increased depression and reduced quality of life scores for patients with idiopathic pulmaonry fibrosis.^35^ The authors postulated that, as the participants had mild disease, it is possible they received palliative care ‘too early’ and discussions regarding prognosis may have worsened symptoms of anxiety and depression. This is a sharp contrast with other studies which proclaim the benefits of early palliative care for people with interstitial lung disease.^20,27^ An earlier US study which evaluated the impact of a nurse-led support group also found that patients in the intervention group rated their quality of life less positively and tended to report more anxiety following the intervention.^36^ These results should be interpreted with caution as although all three studies^32,35,36^ were randomised controlled studies, they included small numbers of participants, were unblinded and were underpowered to detect statistical significance between the groups.

Two studies conducted in Sweden^23^ and Japan^28^ compared end-of-life care for patients with interstitial lung disease with those with lung cancer. Both studies identified that patients with interstitial lung disease were more likely to experience breathlessness at the end-of-life and had reduced access to palliative care services despite their high symptom burden. interstitial lung disease patients required more doses of ‘as required’ medications for symptom management with lower rates of complete relief from breathlessness^23^ and received less opioids compared with those with lung cancer (9.6% ILD vs 32.1% LC; *p* < 0.001).^28^ Patients with interstitial lung disease had a significantly lower mean Good Death Inventory (GDI) score than those with lung cancer (*p* = 0.04) and scored significantly lower in the domains of ‘physical and psychological comfort’, ‘environmental comfort’ and ‘control over the future’.^28^ The authors concluded that patients with interstitial lung disease had a lower quality of death and dying than those with lung cancer.

Overall, symptom control at the end-of-life was poorly assessed by the included studies. Two studies identified a deficiency in documented symptom assessment in interstitial lung disease compared with other chronic lung diseases.^19,23^ Pharmacological treatment was common in the two studies which evaluated symptom management. In the last week of life, 71%–94% patients received opioids and 44%–73% benzodiazepines.^15,26^ A UK retrospective observational study reported that 100% of patients who received opioids or benzodiazepines for relief of dyspnoea had documented benefit from these medications.^24^ However, survey data from Japanese respiratory physicians suggested that opioids were more likely to be prescribed for symptom control in lung cancer than in idiopathic pulmonary fibrosis (*p* < 0.001),^30^ indicating that some physicians may not be familiar with the therapeutic effect of these medications.

### Location of death

Most patients with interstitial lung disease died in a hospital setting,^15,22,24,25,37^ despite evidence that many patients would prefer to die at home or in a hospice.^27^ Data from retrospective cohort studies found that 57%–80% patients with interstitial lung disease died in a hospital setting.^15,22,24,25,37^ Observational studies reported a decrease in the number of patients dying with interstitial lung disease in acute settings over time,^37,38^ which correlates with the trends for other chronic lung diseases. A large UK study found that death in an acute setting was associated with multimorbidity, living in urban areas and socio-economic deprivation,^37^ and correlates with other studies showing that death at home was more likely for patient who were older, married, living in rural areas and who had higher level of education.^25,38^

A Canadian study found that earlier initiation of palliative care increased the number of patients with interstitial lung disease who died in their home or hospice.^27^ This study reported 62% of patients died in a home or hospice environment and those who did were older, more likely to be male and more likely to have idiopathic pulmonary fibrosis. Importantly, this study reported that 61 of 92 patients had their preferred place of death ascertained, which was home or hospice for 96% of patients. Moreover, 90% of these patients died in their preferred place. A large US study identified that married patients were more likely to die at home^38^; alluding to the carer responsibility that is taken on by relatives and especially partners when a person receives end-of-life care at home. Patients were more likely to spend the last days of life at home if their caregiver engaged with advance care planning discussions.^27^

## Discussion

### Main findings

To our knowledge this is the first review to synthesise the available evidence relating to the quality of end-of-life care in interstitial lung disease. We found that most patients died in hospital and were subject to a high burden of investigations and life-prolonging treatments until a few days before death. However, patients who were given the opportunity to participate in advance care planning indicated that their preferred place of death was at home. Low levels of palliative care referral and advance care planning were reported in many of the included studies, which, along with inadequate training and awareness amongst physicians, may account for the discrepancy between patient’s wishes and outcomes. There was a notable paucity of research which considered symptom control at the end-of-life.

### Comparison with other work

Despite the poor prognosis associated with many forms of interstitial lung disease, this review demonstrated low levels of palliative care referral. Patients were more likely to be referred to specialist palliative care if they lived closer to tertiary centres and were seen more frequently in outpatient clinics.^25^ These results imply a geographical disparity in access to palliative care, which has also been identified in previous work by the North East and Cumbria interstitial lung disease service.^39,40^ Variability in access to specialist palliative care services is also known to be an issue for patients with other chronic respiratory diseases.^41^ Referral to palliative care services is likely to be influenced by healthcare professionals’ familiarity with local services and whether specialist services exist. It is therefore important for healthcare professionals at tertiary centres to familiarise themselves with palliative care services throughout their region and signpost to general practitioners patients who should be on the palliative care register and referred onto local services.

Existing literature has recognised that advance care planning conversations were surprisingly uncommon for patients with chronic lung disease; possibly due to uncertain disease trajectories and ‘ambivalence’ amongst both patients and healthcare professionals to engage in discussions.^42^ Engagement with advance care planning interventions has been associated with a reduction in acute healthcare utilisation for patients with chronic respiratory disease.^43^ This review corroborates this conclusion,^21^ and also identified that patients involved with palliative care and advance care planning conversations were subjected to fewer investigations during their terminal admission.^16^ Admission to hospital under general medicine rather than palliative care increased the investigations towards the end-of-life,^16^ demonstrating that recognition of advanced disease and the need for palliation in outpatient setting influences the end-of-life care that patients receive. Clinicians may find it easier to escalate treatment during an acute admission where prior advance care planning has not been undertaken, rather than have complex discussions during an acute event. Previous work has demonstrated the successful introduction of advance care planning interventions during hospital inpatient admission.^44^ However, it remains important for these discussions to be guided by experienced physicians who recognise ‘turning points’ in the disease trajectory and ideally have an established doctor/patient relationship.^45^

Integration of respiratory and palliative care services has been associated with improved symptom management and reduced acute health care utilisation towards the end-of-life for patients with advanced respiratory disease.^46–48^ This review established that integration of respiratory and palliative care services and close links with community services can improve recognition of patients with interstitial lung disease who would benefit from palliative care and advance care planning conversations.^20,32,33^ However, these studies also highlight the significant time and resources required to deliver interventions, which may therefore only be possible for a small number of patients given the increasing strain on health care resources. Devising ways to target palliative care referrals, for example with Integrated Palliative care Outcome Scale (IPOS),^49^ or a supportive care decision tool^50^ would ensure these resources were available for the most appropriate patients.

### Strengths and limitations

Strengths of this analysis include that, the searches were conducted across five research databases, with review of the reference list of included studies to identify additional relevant studies. The included studies originated from the UK, Europe, North America, Canada, Australia and Japan, providing international relevance of the findings. All included studies were published in the last 10 years, reflecting the contemporary importance of the topic. Palliative care research has frequently focussed on idiopathic pulmonary fibrosis and therefore the inclusion of studies involving other types of interstitial lung disease makes these results more generalisable. The GMC definition of end-of-life^14^ was used which contributed to the inclusion of a broad range of research studies.

The heterogenous nature of the included studies led to a comprehensive overview of the issues affecting end-of-life care in interstitial lung disease. However, variations in methodology, quality and relevance generated difficulties with the integration and synthesis of data and establishing common themes. The heterogeneity of the study outcomes reduced the certainty of results as there were limited studies to support the conclusions in each of the identified themes. Many of the included studies were published by specialist interstitial lung disease services and therefore the results may not reflect the standard of care in areas without these services. Notably, all the randomised controlled trials included in this review were rated ‘poor’ in the quality assessment as they were unblinded and underpowered to detect significant change. This highlights the difficulty in conducting randomised controlled trials focussed on palliative care interventions.

### What this study adds

There is societal consensus that factors such as physical comfort and symptom control should be prioritised when the diagnosis of dying has been made.^51^ This review highlights deficiencies in the documentation of symptom assessment and prescription of medications at the end-of-life for interstitial lung disease compared with patients with cancer^19,23,28^ and found people with interstitial lung disease were more likely to have unrelieved symptoms.^23^ These deficiencies in symptom assessment and management combined with the fact that many patients die in hospital reflects poorly on the quality of end-of-life care received by interstitial lung disease patients. However, most studies which reviewed patients’ symptoms were retrospective and did not use validated symptom scores, and therefore were subject to the limitations of documentation, which may not accurately represent the standard of care that was delivered.

Established literature indicates that the majority of people wish to die in their own home.^52^ Documentation of preferred place of death is important as it demonstrates that end-of-life care preferences have been discussed, which corroborates the quality of care even if the location of death is not achieved due to change in circumstances. There is no evidence that patients with interstitial lung disease have different end-of-life preferences to patients with other diseases and when preferred place of death is ascertained, patients predominantly wish to die at home.^20,27^ Engagement with advance care planning interventions has been reported to increase the likelihood of patients dying at home.^53^ Archibald et al reported 90% accordance with patients’ preferred place of death with a high proportion of patients supported to die in their own home.^27^ This challenges the view that patients with interstitial lung disease will inevitably die in hospital due to the uncertain disease trajectory and management of potentially reversible acute exacerbations. It is important to remember that preferred place of care and death often changes with time and increasing burden of symptoms and requires review alongside disease progression.^52,54^ It is unknown whether patients who die at home have good symptom control towards the end-of-life. However, it is possible that patients choose to be admitted to hospital for end-of-life care due to distressing symptoms of severe breathlessness, anxiety or panic, or the requirement for high levels of oxygen which cannot always be provided in the community.

Although most patients with interstitial lung disease die in hospital, it is not known whether this is their preferred place of care or whether family members feel they died in an appropriate place. An appropriate place of death would enable a ‘safe’ death in respect to the level of medical care required.^55^ There is a clear difference between patients dying in the emergency department or intensive care versus patients who die in a side room on a ward with input from palliative care teams in the hospital.^56^ The latter may allow more time for recommendations from *One Chance to Get it Right*^51^ to be enacted and family members may feel that death was more peaceful. It is also important to consider the psychosocial burden that can result from relatives and informal caregivers providing end-of-life care at home.^57^ The more important question to benchmark the success of palliative care interventions is whether the patient died in an appropriate and safe place, rather than whether they died in their own home.

## Conclusion

This review identified five key factors which influence the quality of end-of-life care provided to patients with interstitial lung disease. The assessment and management of patients’ symptoms is fundamental when considering the quality of end-of-life care. However, there is a paucity of research addressing symptom control for people with interstitial lung disease in the last few weeks and days of life. People with interstitial lung disease who have discussions about advance care planning mainly indicate their preferred place of death is home or hospice and very few wish to die in an acute care setting. There is evidence that with early palliative care involvement and advance care planning interventions patients can be supported to die at home. Little is known about the appropriateness or ‘safety’ of dying from interstitial lung disease in either an acute setting or at home. Future research should consider symptoms control at the end-of-life and association with location of death.

## Supplemental Material

sj-pdf-1-pmj-10.1177_02692163211059340 – Supplemental material for Which factors influence the quality of end-of-life care in interstitial lung disease? A systematic review with narrative synthesis Supplemental material, sj-pdf-1-pmj-10.1177_02692163211059340 for Which factors influence the quality of end-of-life care in interstitial lung disease? A systematic review with narrative synthesis by Evelyn Palmer, Emily Kavanagh, Shelina Visram, Anne-Marie Bourke, Ian Forrest and Catherine Exley in Palliative Medicine

sj-pdf-2-pmj-10.1177_02692163211059340 – Supplemental material for Which factors influence the quality of end-of-life care in interstitial lung disease? A systematic review with narrative synthesis Supplemental material, sj-pdf-2-pmj-10.1177_02692163211059340 for Which factors influence the quality of end-of-life care in interstitial lung disease? A systematic review with narrative synthesis by Evelyn Palmer, Emily Kavanagh, Shelina Visram, Anne-Marie Bourke, Ian Forrest and Catherine Exley in Palliative Medicine

sj-pdf-3-pmj-10.1177_02692163211059340 – Supplemental material for Which factors influence the quality of end-of-life care in interstitial lung disease? A systematic review with narrative synthesis Supplemental material, sj-pdf-3-pmj-10.1177_02692163211059340 for Which factors influence the quality of end-of-life care in interstitial lung disease? A systematic review with narrative synthesis by Evelyn Palmer, Emily Kavanagh, Shelina Visram, Anne-Marie Bourke, Ian Forrest and Catherine Exley in Palliative Medicine
