# Supplementary material for: Which factors influence the quality of end-of-life care in interstitial lung disease? A systematic review with narrative synthesis
Source: Palliat Med. 2021 Dec 17;36(2):237–53. doi: 10.1177/02692163211059340 (PMC8894683; doi:10.1177/02692163211059340)
Supplement: sj-pdf-1-pmj-10.1177_02692163211059340 – Supplemental material for Which factors influence the quality of end-of-life care in interstitial lung disease? A systematic review with narrative synthesis [file sj-pdf-1-pmj-10.1177_02692163211059340.pdf]

Randomised Controlled Trials Critical Appraisal Checklist

Title of paper:<br>Authors:				
	Yes	No	Unclear	Comments
Are the results of the study valid?				
1. Did the study address a clearly focused issue?				
2. Was the assignment of participants to interventions randomised?				
3. Were all participants who entered the study accounted for at its conclusion?				
4. (a) Were the participants 'blind' to the intervention being given?				
(b) Were the investigators 'blind' to the intervention being given to participants?				
(c) Were the people assessing/analysing outcomes 'blinded'?				
5. Were the study groups similar at the start of the randomised controlled trial?				
6. Apart from the experimental intervention, did each study group receive the same level of care?				
What are the results?				
7. Where the effects of the intervention reported comprehensively?				
8. How precise are the results?				
9. Do the benefits of the experimental intervention outweigh the harms and costs?				

Will the results help locally?				
10. Can the results be applied to the local population?				
11. Do the results of this study fit with other available evidence?				
12. Would the experimental intervention provide greater value to local patients than any existing interventions?				

Adapted from the checklist for critical appraisal of Randomised Controlled Trials devised by the Critical Appraisal Skills Programme.

Quality appraisal:

• Low risk of bias (all criteria met) = Good

• Moderate risk of bias (one or more criteria unclear) = Fair

• High risk of bias (one or more criteria not met) = Poor
