# Supplementary material for: Which factors influence the quality of end-of-life care in interstitial lung disease? A systematic review with narrative synthesis
Source: Palliat Med. 2021 Dec 17;36(2):237–53. doi: 10.1177/02692163211059340 (PMC8894683; doi:10.1177/02692163211059340)
Supplement: sj-pdf-2-pmj-10.1177_02692163211059340 – Supplemental material for Which factors influence the quality of end-of-life care in interstitial lung disease? A systematic review with narrative synthesis [file sj-pdf-2-pmj-10.1177_02692163211059340.pdf]

Survey Critical Appraisal Checklist

Title of paper:<br>Authors:				
	Yes	No	Unclear	Comments
Are the results of the study valid?				
1. Did the study address a clearly focused issue?				
2. Is the research method appropriate for answering the research question?				
3. Is the method of selection of the subjects clearly described?				
4. Could the way the sample was obtained introduce selection bias?				
5. Was the sample of subjects representative with regard to the population to which the findings referred?				
6. Was the sample size based on pre-study considerations of statistical power?				
7. Was a satisfactory response rate achieved?				
8. Are the measurements (questionnaires) likely to be valid and reliable?				
9. Was statistical significance assessed?				
10. Are confidence intervals given for the main results?				
11. Are all confounding factors accounted for?				
12. Can the results be generalised to the local population?				

Adapted from checklist devised by the Centre for Evidence-Based Medicine.

Quality appraisal:

• Low risk of bias (all criteria met) = Good

• Moderate risk of bias (one or more criteria unclear) = Fair

• High risk of bias (one or more criteria not met) = Poor
