# Supplementary material for: Which factors influence the quality of end-of-life care in interstitial lung disease? A systematic review with narrative synthesis
Source: Palliat Med. 2021 Dec 17;36(2):237–53. doi: 10.1177/02692163211059340 (PMC8894683; doi:10.1177/02692163211059340)
Supplement: sj-pdf-3-pmj-10.1177_02692163211059340 – Supplemental material for Which factors influence the quality of end-of-life care in interstitial lung disease? A systematic review with narrative synthesis [file sj-pdf-3-pmj-10.1177_02692163211059340.pdf]

Cohort Study Critical Appraisal Checklist

Title of paper:<br>Authors:				
	Yes	No	Unclear	Comments
**Are the results of the study valid?**				
1. Did the study address a clearly focused issue?				
2. Was the cohort recruited in an acceptable way?				
3. Was the exposure accurately measured to minimise bias?				
4. Was the outcome accurately measured to minimise bias?				
5. (a) Have the authors identified all important confounding factors?				
(b) Have they taken account of the confounding factors in the design and/or analysis?				
6. (a) Was the follow up of subjects complete enough?				
(b) Was the follow up of subjects long enough?				
**What are the results?**				
7. What are the results of this study?				
8. How precise are the results?				
9. Do you believe the results?				
**Will the results help locally?**				
10. Can the results be applied to the local population?				
11. Do the results of this study fit with other available evidence?				
12. What are the implications of the study for practice?				

Adapted from checklist devised by the Critical Appraisal Skills Programme.

Quality appraisal:

• Low risk of bias (all criteria met) = Good

• Moderate risk of bias (one or more criteria unclear) = Fair

• High risk of bias (one or more criteria not met) = Poor
